# Fuzzy Logic Analysis of Kinase Pathway Crosstalk in TNF/EGF/Insulin-Induced Signaling

**DOI:** 10.1371/journal.pcbi.1000340

**Published:** 2009-04-03

**Authors:** Bree B. Aldridge, Julio Saez-Rodriguez, Jeremy L. Muhlich, Peter K. Sorger, Douglas A. Lauffenburger

**Affiliations:** 1 Center for Cell Decision Processes, Cambridge, Massachusetts, United States of America; 2 Department of Biological Engineering, MIT, Cambridge, Massachusetts, United States of America; 3 Department of Systems Biology, Harvard Medical School, Boston, Massachusetts, United States of America; University of Illinois at Urbana-Champaign, United States of America

## Abstract

When modeling cell signaling networks, a balance must be struck between mechanistic detail and ease of interpretation. In this paper we apply a fuzzy logic framework to the analysis of a large, systematic dataset describing the dynamics of cell signaling downstream of TNF, EGF, and insulin receptors in human colon carcinoma cells. Simulations based on fuzzy logic recapitulate most features of the data and generate several predictions involving pathway crosstalk and regulation. We uncover a relationship between MK2 and ERK pathways that might account for the previously identified pro-survival influence of MK2. We also find unexpected inhibition of IKK following EGF treatment, possibly due to down-regulation of autocrine signaling. More generally, fuzzy logic models are flexible, able to incorporate qualitative and noisy data, and powerful enough to produce quantitative predictions and new biological insights about the operation of signaling networks.

## Author Summary

Cells use networks of interacting proteins to interpret intra-cellular state and extra-cellular cues and to execute cell-fate decisions. Even when individual proteins are well understood at a molecular level, the dynamics and behavior of networks as a whole are harder to understand. However, deciphering the operation of such networks is key to understanding disease processes and therapeutic opportunities. As a means to study signaling networks, we have modified and applied a fuzzy logic approach originally developed for industrial control. We use fuzzy logic to model the responses of colon cancer cells in culture to combinations of pro-survival and pro-death cytokines, making it possible to interpret quantitative data in the context of abstract information drawn from the literature. Our work establishes that fuzzy logic can be used to understand complex signaling pathways with respect to multi-factorial activity-based protein data and prior knowledge.

## Introduction

**Figure 1:**
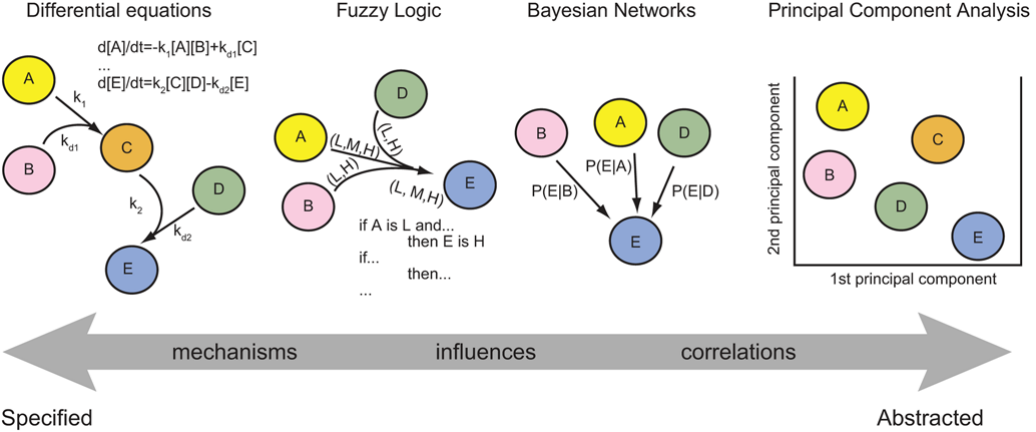
Spectrum of modeling methods. Modeling techniques balance specificity and complexity. Principal component analysis elucidates correlations among network components (A–E) by a linear transformation of the data, resulting in orthogonal principal components. Bayesian networks use conditional probabilities to associate correlations and influences between network components. Fuzzy logic uses rule-based gates and probabilistic representation of input variables to quantify influences and mechanism that regulate network species. Differential-equations models using mass-action kinetics are highly specified defining regulatory mechanism by defining rates of change in network species concentrations.

A variety of modeling methods can be applied to understanding protein signaling networks and the links between signals and phenotypes [1]. The choice of modeling method depends on the question being posed (e.g., mechanistic or phenotypic), the quality and type of experimental data (quantitative or qualitative), and the state of prior knowledge about the network (interaction map or detailed biochemical pathway; Figure 1). Abstract techniques are largely data-driven and aim to discover correlations among signals or between signals and cellular phenotypes [2]–[4]; these methods include principal component analysis (PCA) and partial least-squares regression (PLSR). Mechanistic differential equation-based models, in contrast, are highly specified and dependent on extensive prior knowledge about components and their interactions, but have the advantage that they capture temporal and spatial dynamics at the level of individual reactions [5]–[9]. Between these extremes, modeling methods such as Bayesian statistics, hidden Markov models, and logic-based models have been used to construct graph-based representations of influences and dependencies among signals and phenotypes based on experimental data [10]–[18]. An advantage of these methods is their applicability to situations in which mechanistic information is incomplete or fragmentary but the notion of a network of interacting biochemical species is nonetheless informative. Moreover, logic-based models use natural language to encode common logical statements such as “if the kinase is not active or the phosphatase is overexpressed, the substrate is not phosphorylated”. Logic-based models are commonly depicted as edge-node graphs in which interactions among species occur at nodes, with gates specifying the logic of the interactions based on a set of specified rules. The identities of the gates are typically determined based on prior knowledge or experimental observables and the input-output relationships of each gate inferred from experimental data [11], [12], [19]–[24].

**Figure 2:**
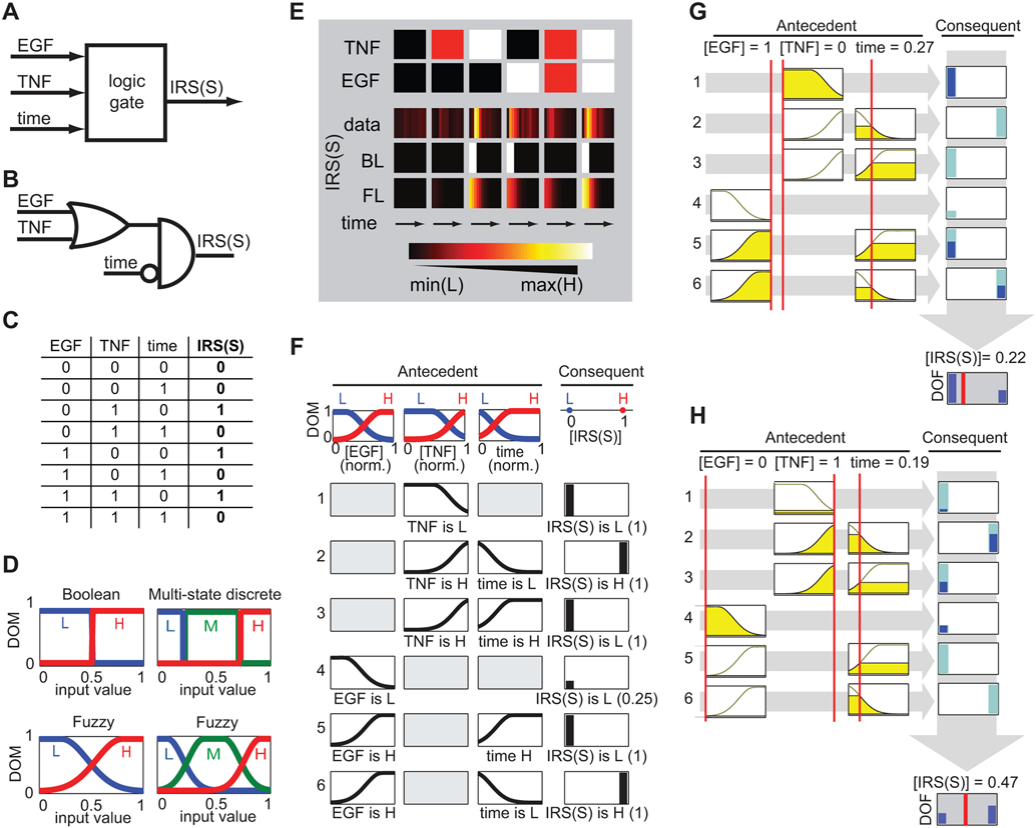
Fuzzy logic modeling process. As an example, local logic gate construction is illustrated for IRS(S) (IRS phosphorylation at serine 636). (A) Logic-based models use incoming edges to contain activity level of input or regulatory network species (for IRS(S), the inputs were TNF, EGF, and time) with the logic gate at the node that performs the logic operation to update output signal (IRS(S)). (B) A Boolean logic gate for IRS(S) could be represented in terms of the logic statement “(TNF or EGF) and (NOT(time))”, represented here in schematic form where the top shape is an “OR-gate” the circle is a “NOT” operation, and the lower left shape is an “AND-gate”). (C) The truth table for the logic in (B) states the output of IRS(S) (0 for off or 1 for on, in bold) based on the input state. (D) To set up a FL gate, the first step is to assign membership functions (MFs) to the input variables (“TNF”, “EGF”, and “time”). In this example, each input variable has two or three membership functions (“L”, “M”, and “H” representing low, medium, and high states, respectively). An MF relates an input value to that state's degree of membership (DOM). MFs for Fuzzy and Boolean (2 MFs)/discrete multi-state (>2 MFs) logic forms are illustrated with the same state thresholds. (E) The simulations from the Boolean logic gate shown in B–C is compared to experimental data and the Fuzzy logic gates specified in F below (see Figure 5A for the experimental and simulation conditions). The BL gate is not able to model intermediate state for smooth transitions, and simulations of the FL gate better fit the data as compared to the BL gate. (F) To set up a FL gate, the MFs for the inputs and the constant values for the outputs are defined. For simplicity, we use normalized input and output values. Next, logic rules are listed as “if A (the antecedent), then B (the consequent)” using the input and output states as descriptors. Weights between 0 and 1 are assigned to each rule (indicated in parentheses), which is helpful for rules that should have minor influence (e.g. rule 4). The rules for IRS(S) are each graphically listed with the outline of the membership functions specified for that rule's antecedent. Inputs not considered for an antecedent are indicated by a light gray box. The consequent for each rule is indicated by a bar whose height is proportional to the rule weight. We do not depict FL rules in a truth table because a row is not necessarily unique in FL (c.f. (C)). (G–H) Two input scenarios are presented to illustrate FL gate computation (horizontal gray arrows) and defuzzification (vertical gray arrow). The amount of color filled in (yellow for inputs and blue for output) is representative of the DOM (for inputs) or degree of firing (DOF) given the input values (for outputs). The input values are listed on the top and indicated graphically by the vertical red lines. For example in scenario 1, rule 1 fires (full dark blue bar) because the antecedent (TNF is H) has a high DOM (filled in yellow). The firing strength of the rule is the minimum of the antecedents; therefore, rule 2 does not fire because while time has low DOM to L (∼.4) and the DOM of time to H is near zero. To defuzzify (resolve the output value given a set of firing rules), an average is computed from the output values of each rule weighted according to both firing strength and rule weight (see Methods). The bottom row in the consequent column shows the aggregated outputs and the small red line is the defuzzified or final, value. The scenario illustrations were adapted from the “rule viewer” in Matlab's Fuzzy Logic Toolbox.

Among logic-based methods, the simplicity of Boolean models makes them attractive as a means to render biological networks. For example, a discrete-state representation of the level of phosphorylation of insulin receptor substrate 1 (IRS-1) at serine 636 (IRS(S)) might use three input edges for time, TNF and EGF (see below), one output edge for IRS(S), and one logic gate (where “1” means present or active, and “0” absent or inactive; Figure 2A). Time is included as an input variable to enable the representation of transient responses, following cytokine treatment, for example. In Boolean logic, interactions among inputs are cast as combinations of elementary “AND”, “OR”, and “NOT” gates that generate logic rules such as “(EGF OR TNF) AND (NOT(time))” and are most easily specified using truth tables (Figure 2B–C). Truth tables consist of lookup values for the outputs (consequent value) based on all possible combinations of input values (antecedents). Despite the appeal of Boolean models a two-state “on-off” representation of many biological signals is quite unrealistic [25]–[27].

In this work, we propose fuzzy logic (FL) as an approach to logic-based modeling with the easy interpretability of Boolean models but significant advantages [28] including the ability to encode intermediate values for inputs and outputs. We show that FL can encode probabilistic and dynamic transitions between network states so as to create simple and fairly realistic depictions of cell signaling networks [20]–[23], [29]–[31]. A key advantage of logic-based approaches, also exemplified by FL, is the ability to construct models *ad hoc* based on knowledge of network topology and data [32]–[36]. Reverse engineering models from data is an alternative and complementary approach, which is less biased by *a priori* knowledge and assumptions, and is particularly useful for identifying plausible topology and parameterization given quantitative data gathered under several perturbations. Here, we focused on building models by hand because our goal was to test whether FL methods could be adapted to test *a priori* knowledge and hypotheses against data to refine our understanding of the network and generate testable hypotheses. We complement our initial model with model optimization to compare the effects of fuzzification.

Several means to refine Boolean models have been described, including kinetic logic and the closely related piecewise-linear differential equations systems [22],[37],[38]. Some of these extensions rely on a differential equation system coupled to the Boolean network to handle continuous variables. The resulting models share common steady-state behavior with the underlying Boolean system (which is especially useful, for example, in development and cell cycle studies) [39], but take longer to simulate since they involve solving differential equation systems rather than look-up tables. Like fuzzy logic, dynamic Bayesian networks (BN) (and the related probabilistic Boolean networks [40]) are able to handle data in a non-discrete fashion, and have been used extensively to reverse engineer biological networks and to model uncertainty in signaling networks [4],[13],[41],[42]. However, the theoretical foundations are very different from those of FL: BNs are based on probability distributions, in contrast to membership functions in FL (see below). Accordingly, the interpretation is also significantly different: BNs assign a probability that a particular interaction exists (with pre-defined weights), while FL assigns rule weights to describe the interactions thought to be present. We argue that FL models represent a useful addition to the set of mathematical methods available for analyzing complex cellular biochemistry.

The death-survival decisions made by mammalian cells in response to environmental stimuli, such as those examined in this paper, are mediated by the integrated activities of multiple receptor-dependent and cell-intrinsic processes that coordinate opposing pro- and anti-apoptotic signaling. We have previously described a “cue-signal-response” (CSR) compendium of protein signals and phenotypic responses in HT-29 human colon carcinoma cells treated with combinations of tumor necrosis factor-α (TNF), epidermal growth factor (EGF), and insulin [43]. The compendium includes ten measurements of protein modification states (phosphorylation and cleavage) and kinase activities for four proteins downstream of TNF, EGF and insulin receptors collected over a 24 hr time period in biological triplicate. To date we have used PLSR to predict the phenotypic consequence of perturbing the signaling network [44] and PCA to identify autocrine feedback circuits [45].

In this paper we explore the ability of a manually assembled multi-state FL model to encode the dynamics of a complex intracellular signaling network. We find that key features of FL, such as non-discrete input-output relationships (membership functions – see below) and the possibility that more than one relationship can be invoked at the same time results in a remarkably intuitive representation of biology. It was therefore possible to generate new biological insight into the regulation of IKK (IkB kinase) and MK2 (mitogen-activated protein kinase-activated protein kinase 2) kinases simply by inspection of the model. A closer fit between the FL model and data could presumably be achieved by automated regression. As a step in this direction we converted the multi-state FL model into a 2-state FL model that could be calibrated against data. The calibrated 2-state FL model exhibited a better fit to data than a discrete model having the same degrees of freedom. The calibrated 2-state FL model also exhibited a better fit than the manually assembled multi-state FL model, but only at the cost of less interpretability. Overall we conclude that manual assembly of FL models is an effective means to represent signal transduction and derive biological insight; development of new approaches to automated model fitting should also make FL models effective tools for prediction.

## Results

Prior knowledge of signal transduction biochemistry was used to assemble a topological framework covering all experimental observables in the CSR dataset and logic then added using an adaptation of the FL toolbox in Matlab. Once gates were specified, a global model was constructed by connecting FL gates together and the behavior of the global model was evaluated with respect to goodness of fit to data. Specifically, FL gates were used to model changes in protein concentrations or their states of modification. Each protein in the network was associated with a single FL gate whose inputs were specified by the framework topology; the effect of the inputs on the activity or concentration of the protein represented by the FL gate was then determined using prior literature knowledge and data. Specifying the precise operation of each FL gate involved two distinct concepts: definitions that assigned to each input a *membership* to descriptive *classes* (*states* such as “low”, “medium”, and “high”), and logic *rules* that related these input classifications to a specific output.

Working with FL models involves manipulating logic gates based on several adjustable parameters: (i) *Membership functions* (MFs) are used to assign values of inputs to a descriptive input class. (ii) MFs define the *degree of membership* (DOM) that quantifies the mapping between inputs and MFs and is always between 0 (no membership) and 1 (full membership). Fuzzy logic is so-named because inputs can have non-zero DOM to more than one MF, unlike discrete-state logic in which MFs and DOMs only take on values of 0 and 1 [28],[46]. Figure 2D illustrates example MFs for Boolean and fuzzy logic models. (iii) The steepness of the membership functions is parameterized by the *degree of fuzziness* (note that Boolean logic models have a degree of fuzziness of 0). (iv) Logic *rules* relate the input state to the output state. In doing so, these rules encode how the input proteins regulate the activity of output protein.

Once the logic rules are established, an FL gate is generated by first *fuzzifying* the inputs, a step that computes the DOM of each input state over the current input values and the pre-specified MFs. The *degree of firing* (DOF), then specifies whether a rule should be used (1) or not (0) as determined from the lowest DOM amongst the antecedents and the *rule weight*, a value between 0 and 1 that allows additional tuning of a rule's importance. In contrast to Boolean logic (BL) gates in which only one rule can fire for any set of input values (that is, only one row in the truth table is applied), FL gates allow multiple rules to fire to varying degrees (as defined by the DOF, Figure 2F). *Defuzzification* is the final step in which the superposition of multiple rules is resolved to determine the output value for the gate. Because of the flexibility of FL gates at the input and output levels, intermediate levels of activity and complex processing functions can be modeled using networks similar in overall structure to familiar BL networks (Figure 2E,G–H). However, flexibility also comes at the cost of additional free parameters; to minimize their numbers we use only a subset of available FL functions. This involves using few intermediate (between 0 and 1) rule weights or membership classes and allowing only one degree of fuzziness for all inputs in a given gate.

### Data for simulation

**Figure 3:**
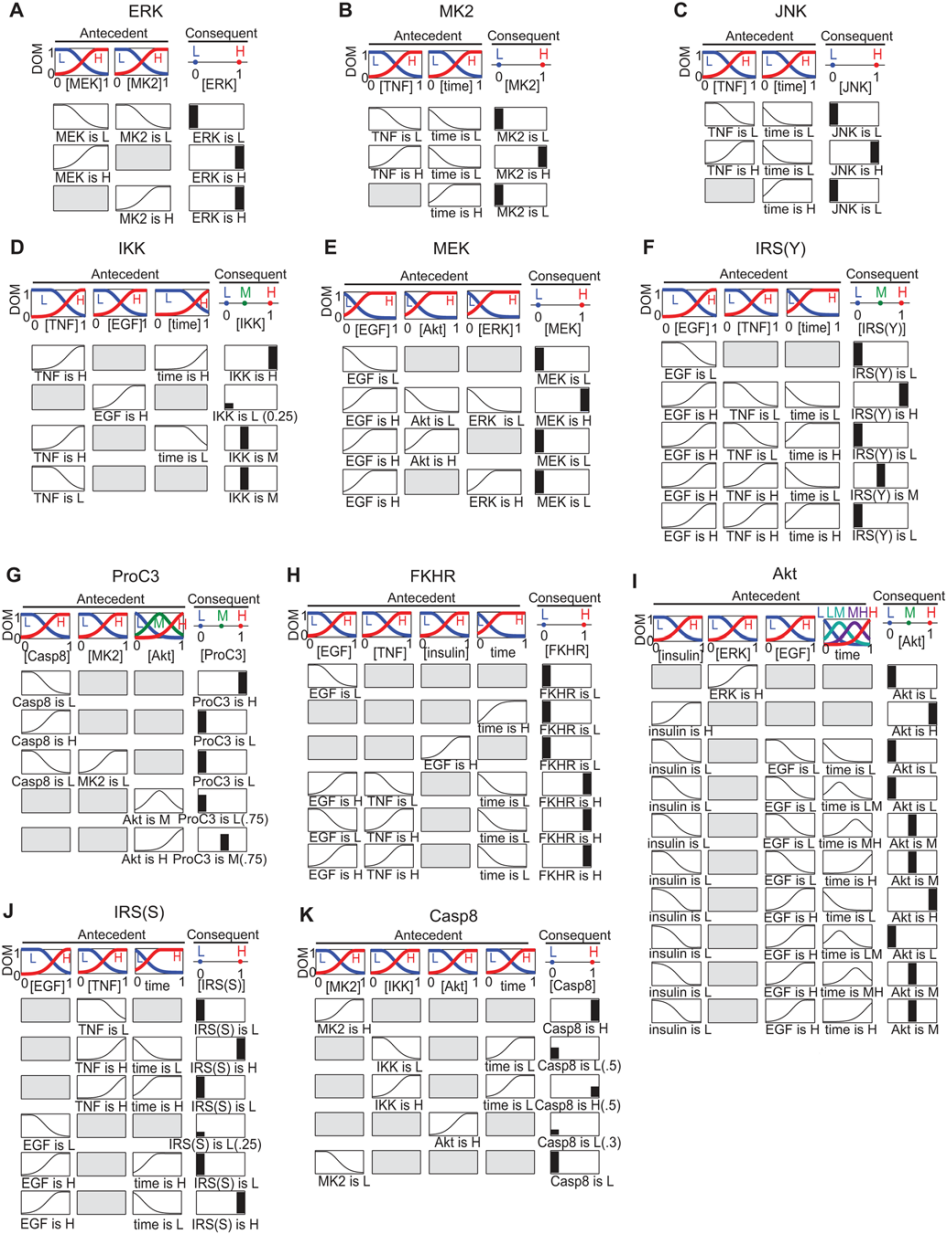
FL gate specifications. Each subfigure depicts the MFs and logic rules for the FL gates: (A) ERK, (B) MK2, (C) JNK, (D) IKK, (E) MEK, (F) IRS(Y), (G) ProC3, (H) FKHR, (I) Akt, (J) IRS(S), and (K) Casp8. The notation is identical to Figure 2, except that rule weights are specified only when they are not 1 and input and output concentrations are normalized (arbitrary units).

**Figure 4 pcbi-1000340-g004:**
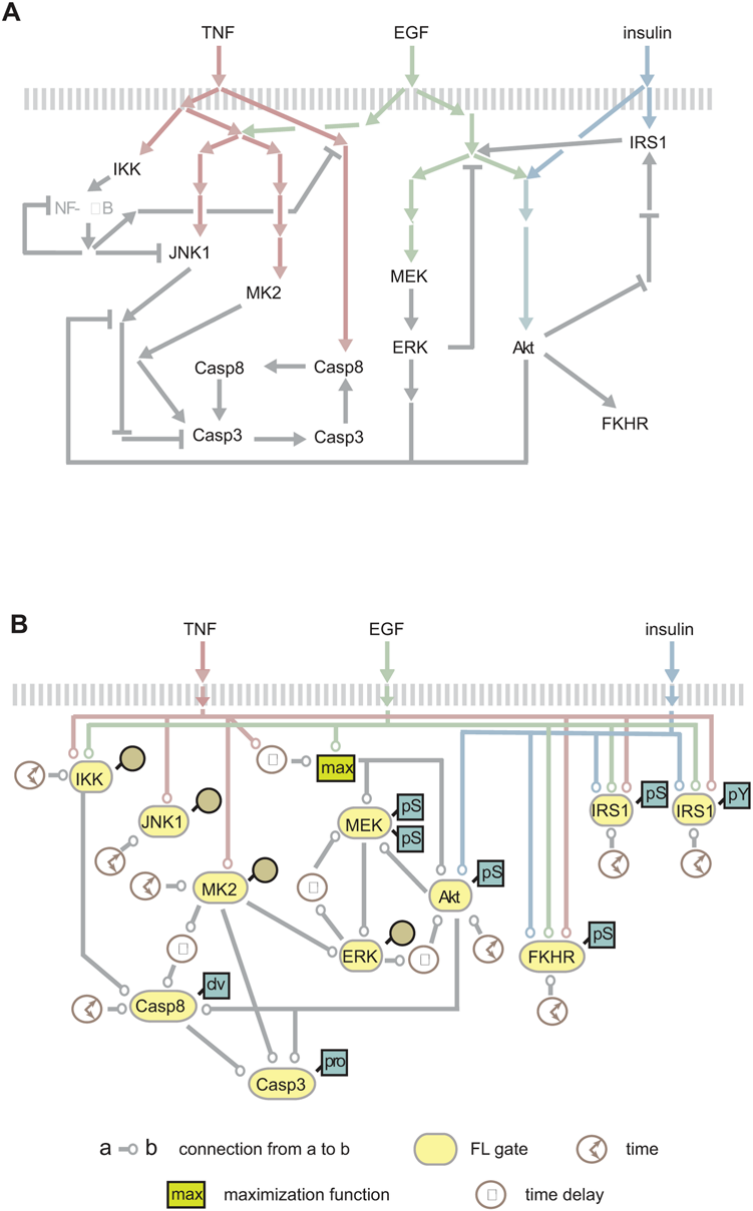
Network diagrams. (A) The original network diagram is adapted from Janes et al. [45] and was used as a starting point to construct the FL gates. Network species whose concentration was measured by Western blot in the data-compendium are notated with a blue square (“pS” for phospho-serine, “pY” for phospho-tyrosine specific antibodies, “clv” for the cleaved form, and “pro” for the uncleaved form). Brown circles mark data compendium proteins measured by kinase assay. (B) This diagram depicts the global FL model, comprised of the 11 local FL gates with time delay and “max” functions. The network topology of the model differs from that of the original diagram.

Working from a normalized heat map of CSR data and the pathway scaffold from Gaudet and Janes et al. (Figures 3–5) [43],[44], gates were manually constructed for signals such as phosphorylation, activation, or total protein levels (Figure 3, Figure 4B). These intracellular proteins in the model include MK2, c-jun N-terminal kinase (JNK), extracellular signal-regulated kinase (ERK), Akt, IKK, Forkhead transcription factor (FKHR), mitogen-activated protein kinase kinase (MEK), IRS-1, cleaved caspase-8 (Casp8), and pro-caspase-3 (ProC3). The first five measurements characterize central nodes in five canonical kinase pathways governing epithelial cell death: FKHR is a transcription factor downstream of Akt; MEK is a kinase directly upstream of ERK; IRS(S) and IRS(Y) represent modifications of insulin receptor substrate (IRS) by insulin receptor; and cleaved-caspase-8 is the active form of the initiator caspase that cleaves caspase-3, an effector caspase responsible for degrading essential cellular proteins, activating CAD nucleases and killing cells.

**Figure 5:**
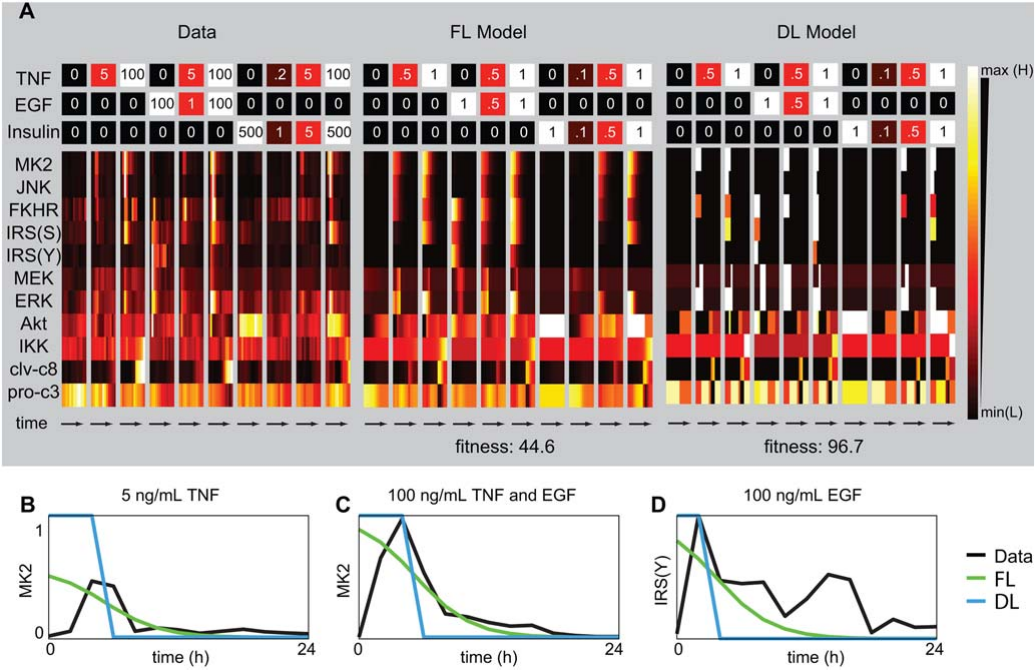
The experimental data compendium and simulation of the global FL model. (A) The left heatmap portrays the averaged normalized data from the experimental compendium [20]. Ten stimulation conditions with TNF, EGF, and insulin (top) are shown with the measurements at 0, 5, 15, 30, 60, 90,120, 240, 480, 720, 960, 1200, and 1440 minutes below. Measurement types (western blot or kinase assay) are indicated in Figure 4A and are described in detail in Gaudet et al. [43]. In the middle, the heatmap shows the results of simulation using the global model under normalized treatment conditions, corresponding with the data compendium shown on the right. Identical simulations of an equivalent discrete logic model (DL, built by changing only the degree of fuzziness from the FL model and leaving the rules and MF thresholds unchanged) are shown on the left (see Methods). The cytokine treatment concentrations are marked directly on the heatmap in ng/mL for the data and arbitrary units for the models. See Figure S5 for an alternative depiction of the data and simulation results. The FL and DL models have fitnesses of 44.6 and 96.7, and normalized fitnesses of 0.035 and 0.076, respectively. (B–D) Simulation and data time courses are plotted for three treatment conditions to highlight cases where the FL model fit the data better than the DL model (B), where both models have similar performance (C), and both models fail (D).

### Assembling a fuzzy logic gate

To illustrate how FL was used to model an intracellular signaling protein, consider the gate describing control of IRS-1 phosphorylation at serine 636 (IRS(S)) by EGF and TNF (Figure 2F–H). For IRS(S), the inputs were TNF concentration, EGF concentration, and time, and the output was the level of IRS(S) phosphorylation. The input and output activities were normalized between 0 and 1 for simplicity. For example, in the IRS(S) gate, TNF concentrations of 0, 5, and 100 ng/mL were normalized to 0, 0.5, and 1 as input values to the FL gate (see Methods). Because we do not explicitly model biochemical processes such as receptor downregulation that make signals transient, some of the FL gates had an input corresponding to time (more generally, this approach makes it possible to model dynamical processes using a logical framework). In the CSR data, “low” times refer to early signaling responses (0–2 hr) while “high” times refer to late signaling events (2–24 hr). Membership functions were defined to transform input values to the DOM for each state. For IRS(S), the EGF input has low (L) and high (H) states (Figure 2F). When normalized EGF activity was ∼0, the gate assigned a high (∼1) DOM to L and low (∼0) DOM to H. As the EGF activity increased to 0.5, DOM = 0.5 for both L and H. The output level classes (L and H) were treated as constants (see Figure 2F); MFs were unnecessary here because gradation of the output was obtained during defuzzification (see below). Once the membership functions had been defined, logic rules were listed as “if A (the antecedent), then B (the consequent)” statements using the inputs and output states as descriptors; e.g., rule 2: if TNF is H and time is L then IRS(S) is H (Figure 2F). Each rule had an associated weight factor between 0 and 1, which was used to quantify the relative importance of the rules.

To compute the output of a gate for a given set of input values, we first fuzzified the input variables (see two examples in Figure 2G–H and described in text below). Next, each rule was evaluated, and a DOF was calculated as the minimum of the DOMs for the inputs and the rule weight [28],[46]. Finally, the outcomes of each rule fired were resolved into a net output value by defuzzification that involved computing the weighted average of the rule consequences (see Methods). By way of illustration, consider the two input value scenarios in Figures 2G–H. In scenario 1 (Figure 2G), EGF = 1 (that is DOM to MF H = 1), TNF = 0 (DOM to MF L = 1), and time = 0.27 (DOM to MF L = 0.4 and H = 0.6). Rule 1 fired entirely (output IRS(S) was L) while rules 5 and 6 fired partially because time had partial membership to L and H (antecedents for rules 6 and 5, respectively); rules 2, 3, and 4 did not fire to a meaningful extent. Combining all these, the aggregate gate output was ∼0.2, an intermediate value between the full L output from rule 1 and the partial H output from rules 5 and 6. In contrast, scenario 2 (Figure 2H) shows a condition (EGF = 0, TNF = 1, time = 0.19) that led to full firing of rule 4 (though this rule has a weight of 0.25), partial firing of rules 2 and 3, and negligible firing of rules 1, 5, and 6. The aggregate gate output in this case was ∼0.5.

### Features of various logic gates

To model CSR data [43], eleven gates were constructed, each comprising 2–4 inputs, 2–4 MFs per input, and 2–3 outputs (see Figure 3). The precise structure of each gate was based on the network scaffold, as described above (Figure 4A). We aimed for as few inputs, rules, and MFs as possible while still allowing a good fit to data. The parameter values for MFs and rules were fit manually to data but future implementation of machine-learning algorithms or automated fitting would improve the speed and accuracy of the process (see below). By way of illustration consider the JNK and MK2 pathways, which are activated by stress and cytokine treatment and are thought to be co-regulated following EGF or TNF treatment (Figure 4A, [47]). During the course of constructing gates for JNK and MK2, we found that the data could be modeled without knowing whether or not cells had been treated with EGF or insulin, suggesting that activation of JNK and MK2 was independent of ligand addition (Figure 3B–C). In some cases, gates based on the pathway scaffold were insufficient to yield a reasonable fit to data and major changes were required in the number and/or types of inputs. For example, IRS-1 is the canonical adapter protein downstream of the insulin receptor, though some of its many phosphorylation sites are also substrates of other receptor kinases, including EGFR [48]. In modeling IRS-1 phosphorylation at two sites, tyrosine 896 (IRS(Y)) and serine 636 (IRS(S)), we observed that both were regulated by TNF and EGF but not by insulin (Figure 3F and 3J). The rules indicate that both TNF and EGF treatment induce S636 phosphorylation while TNF inhibits EGF-induced phosphorylation at Y896 (see Text S1).

During construction of an FL gate for Akt, we included inhibitory crosstalk from ERK to Akt because it has been observed in several experimental settings [49]–[51]. The introduction of crosstalk greatly simplified the rule-base of the Akt gate, suggesting that this crosstalk exists in HT-29 cells (Figure 3I). The mechanistic basis of crosstalk is not fully, and our model includes a short time delay from ERK to the Akt gate input. Negative crosstalk from the ERK to Akt pathways may be the mechanism by which TNF inhibits Akt phosphorylation upon insulin treatment, as observed by Gaudet et al. [43].

### FL network modeling

A model with four inputs (TNF, EGF, insulin, and time) and describing the full CSR dataset was constructed by joining together individual gates specified using the approach described above. Time delays were incorporated to model slow processes such as the induction of transforming growth factor-α [TGF-α] by TNF stimulation [45]. TGF-α, which acts in an autocrine fashion (not shown) was united with the EGF input by taking the maximum value across both signals at each point in time (using the “*MAX*” function), as these ligands bind the same receptor and both affect MEK and Akt FL gates (Figure 4B). To compute model output, a simulator stepped through small time steps, updating inputs to each gates at successive steps (see Methods); model state was then recorded at twelve equal time intervals corresponding to the experimental time points.

Figure 5A depicts heatmaps of the CSR dataset and the FL model, and shows that our FL model recapitulated most major features of the CSR dataset across ten cytokine combinations (Figure 5A). For most inputs, the difference between simulation and experimental data were small, averaging ∼2.2%, over the entire CSR data set (as defined by the root mean square deviation normalized by the mean of the data). Common to all predicted signals was the absence of a delay in activation after cytokine stimulation (Figure 5). To model this delay would require an additional MF for several gates, a feature we omitted for simplicity. It was also challenging to model FKHR phosphorylation. Even though Akt is known to regulate FKHR [52], the model did not effectively match data when Akt was the sole input to the FKHR gate; thus, we modeled FKHR as having inputs from TNF, EGF, insulin, and time (Figure 3H). This suggests that in HT-29 cells, FHKR is subject to more complex regulation than simply activation by Akt.

**Figure 6:**
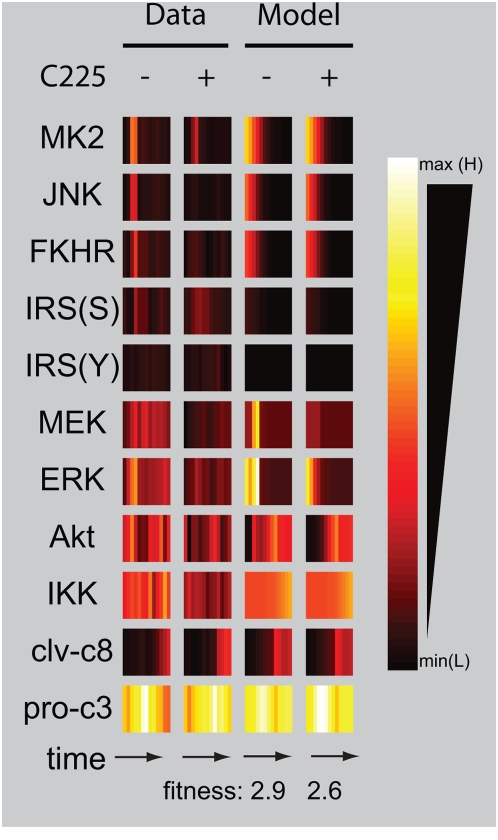
Model prediction of C225 interference with TNF-stimulated signaling. A heatmap depicts the experimental and FL-model predicted response of cells co-treated with 5 ng/mL of TNF and 10 µg/mL of C225 (an antibody that interferes with ligand binding to the EGF receptor), as compared to TNF alone. The model fitness without and with C225 are 2.9 and 2.6, respectively.

One way to evaluate the performance of a model is to ask whether it can correctly predict data that are not part of the training set. Data describing the response of HT-29 cells to co-treatment with TNF and C225, an antibody that blocks ligand binding to the EGF receptor, was not used to assemble the multi-state FL model. We therefore asked whether the FL model could predict the effect of C225 as compared to treatment with TNF alone. Because EGFR is activated both by exogenous EGF and autocrine TGF-α (whose production is induced by TNF [45],[53]) we modeled the effect of C225 addition by disabling the *MAX* function downstream of TNF and EGF (recall that this gate is present to model activation of EGFR not only by exogenous EGF but also by TNF-dependent release of TGFα, which acts in an autocrine manner). The model correctly predicted that cotreatment with TNF and C225 would reduce Akt, MEK, and ERK signals as compared to treatment with TNF-alone (“−“ vs “+” C225 in Figure 6). However, the model did not predict decreases in MK2 and JNK signaling because the MAX function downstream of EGFR activity was not connected to the MK2 and JNK pathways, which are thought to be downstream of TNF but not TGFα or EGF stimulation [45]. We can reinterpret our initial assumptions that TGFα signaling only affects Akt and ERK. The other MAP kinases measured (MK2 and more noticeably JNK) exhibited less activation in the presence of C225. Likewise, late IKK signaling was decreased and slightly more caspases were cleaved compared to C225 alone, but these effects were not predicted by our model. The discrepancy between the model and data suggest that MK2, JNK, and IKK are activated in part by TNF via TGFα by either a direct effect of EGFR or through crosstalk with the Akt and ERK pathways. Our model enabled us to predict some of the effects of C225 in interfering with TNF signaling while providing context to revise our understanding of TNF-induced signaling through EGFR in the MK2, JNK, and IKK pathways

### Towards a method for optimizing gates

**Figure 7:**
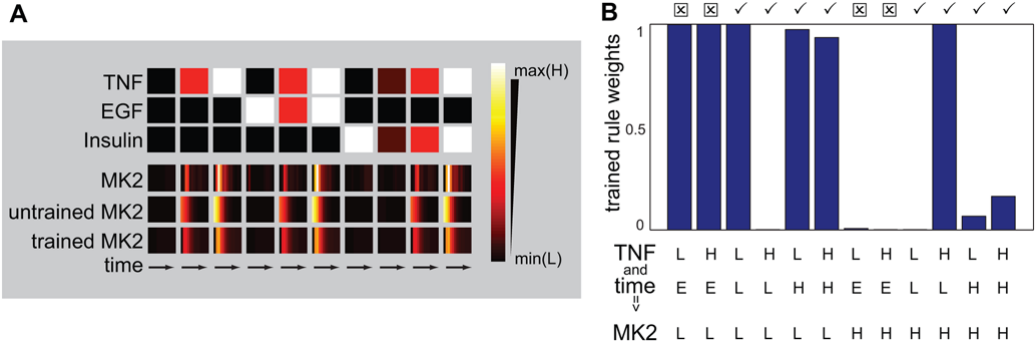
Fitting MK2 rule weights. (A) A heatmap depicts the data, untrained model (Figure 3B), and trained model time courses for MK2. (B) The regressed rule weights are plotted for the 12 candidate rules. The rules are indicated in tabular format; the first two rows describe the state of the inputs, TNF and time, and the last row is the output MK2 state. L and H represent low and high states, and E is the state describing the early response lag. Symbols above the plot show whether the rules were present (✓) or not applicable (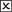) in the untrained model.

In the work described above, logic rules and membership functions for each gate were established manually. A better approach is to use training to optimize the weights of all possible rules in a gate by minimizing the sum of the squared differences between the experimental data and local model output (see Methods). Following optimization, logic rules that are supported by the data should have weights near 1, while poorly-supported rules should have weights near 0. We tested the fitting algorithm on the MK2 gate. For such a gate, which has two MFs each for the two inputs (TNF and time) and the output (MK2 activity), 2^3^ = 8 explicit rules are possible. MK2 data from the 10 cytokine treatment conditions were used to optimize a vector containing the 8 rule weights. Our initial optimization attempt failed because time-dependent MFs were not parameterized so as to capture rapid increases in signals following cytokine treatment. We had implicitly ignored this discrepancy when fitting the model by hand. To improve the automated fitting procedure, an additional MF for time was included to represent immediate-early responses, increasing the number of candidate rules to 12. Optimization yielded a gate with a good fit to data using only six rules with weights near one (Figure 7A). These six rules were identical to those assembled manually with the exception of the new rule needed to represent immediate early signaling (Figure 7B). To test FL gate regression with more rules, we applied the algorithm to the same MK2 data using one additional membership function (for medium activity levels) and compared it to an untrained model using the same MFs. The training process created several rules that were nearly identical to those introduced manually as well as several new ones (Figure S1). The MK2 test case suggests that it is possible to optimize rule weights as a means to fit logic rules without bias and is a first step towards a more rigorous approach to logic-based modeling.

### Comparing fuzzy and discrete logic

To compare FL and discrete models we converted our FL model to a multi-state discrete model (DL) by leaving the rules, rule weights and MF thresholds the same and changing the degree of fuzziness of the MFs so as to make the model discrete (Figure 2D, Methods). Resulting FL and DL models are therefore identical except in a single global parameter (the degree of fuzziness) making direct comparison possible. More than one rule could fire at the same time in both the FL and DL model, making defuzzification necessary in both (see Figure S2). Thus, the DL model was not a conventional Boolean model.

To measure the goodness of fit of FL and DL models, we computed the sum of squared differences (RSS) and normalized RSS (see Methods). The FL model consistently exhibited a better fit to the data than the DL model (absolute deviation of 44.6 and 96.7, and normalized deviation of 0.035 and 0.076, respectively). When we compared simulated and actual data we observed cases in with FL models were better than DL models, cases in which they were similarly effective and cases in which neither did a good job in fitting data. In general, DL models were less effective than FL models in capturing intermediate activity levels (Figure 5B). For example, in the DL model ERK activity alternated between low and high while in the FL model ERK activity was graded, as it was in experimental data (Figure 5A). More striking breakdowns between the DL model and data were observed for IRS(S), JNK and Akt, (Figure 5A). For IRS(S) transient activation was missing from in the model for 1 of 5 cytokine treatments and for JNK it was missed for 3 of 6 treatments However, DL models effectively capture step functions and they are therefore well suited to sharp transient signals (Figure 5C). We also observed cases where both models failed to fit the data, especially when two peaks of activity were observed (Figure 5D). This failure to fit data could be remedied by adding more input states for time and by altering the rules (Figure S2).

To ensure that the superior fit of the FL model (as compared to the DL model) was not biased because the FL model (and not the DL model) was manually assembled, we independently optimized simplified FL and DL models. We performed a global optimization with 8-fold cross-validation of the rule weights in 2-state FL and DL models (see below, Methods, and Figures S2). These models contain two states for each input and the output in every gate. Optimization of the 2-state FL model improved the estimated error compared to the 2-state DL model (with averages and standard deviations of 0.030±0.005 and 0.040±0.006, respectively, using a normalized fitness measure (see Methods and Figure S2). Additionally, we converted the 2-state DL model to BL by converting the rule weights to a binary value (0 or 1). We repeated the optimization but over binary rule weights for the BL and FL 2-state models. The cross-validated error of the binary-weighted FL model was ∼50% lower as compared to the BL model (0.056±0.01 and 0.083±0.01, respectively). We therefore find that a standard Boolean model has poorer performance than the discrete model (DL) studied here (see Figure 2E, Discussion, and Figure S2). The improved ability of the DL model (as compared to the BL model) to predict data following optimization on a training set suggests that continuous rule weights confer noticeable flexibility to the models.

### Biological predictions

As a second means to evaluate the multi-state FL model we looked for new and potentially testable biological insights (see also Text S1).

#### Mk2 and Erk co-regulation

The CSR dataset included information on three MAPK pathways. JNK and p38 respond to TNF and, following cytokine treatment, are jointly regulated by the upstream kinases MEKK1-4 [47]. Since MK2 is a substrate of p38, it was not surprising to see a close correlation in the FL model between JNK and MK2. MEK is the immediate upstream activator of ERK [47], but the fit to ERK dynamics was much better if both MEK and MK2 were included as inputs to the ERK gate; under these circumstances, only five simple rules were required to capture ERK dynamics (Figure 3A, Figure 5A). Moreover, we judged MK2 to be superior to TNF, EGF, and insulin as an input to the ERK gate because the rule-base was smaller. In the final formulation, MK2 “OR” MEK positively influence ERK. The unexpected involvement of MK2 in ERK regulation suggests either that MK2 regulates ERK in an indirect or direct manner, or that MK2 is tightly correlated with an as-yet unidentified ERK regulator. In previous PLSR modeling, we had observed a role for MK2 in cell survival and the current data suggest that ERK may be an effector of MK2 survival functions [44]. MK2 has previously been reported to regulate TNF and TGF-β expression, two ligands that regulate ERK by engaging cell-surface receptors [54], and it is possible that the action of MK2 on ERK is autocrine-indirect. However the time-independence of the interaction in FL model is suggestive of a more direct link.

#### EGF-stimulated inhibition of IKK

TNF receptor (but not EGF receptor) is a potent activator of the canonical Nf-κB pathway, which involves IKK (Figure 4A, [55],[56]). However, IKK can be activated by EGF in some cell types (e.g. estrogen receptor negative breast cancer cells) [57]. In building the FL gate for IKK activity, we were surprised to find that fit to data was improved by adding a simple rule: “If EGF is H then IKK is L (weight 0.25)” (Figure 3D). The necessity of this rule suggests that EGF is a weak, but not insignificant *inhibitor* of TNF-mediated IKK activity.

We have previously reported that in HT-29 cells, TNF induces a complex autocrine cascade in which TNF-induced TGF-α secretion leads to EGF receptor activation and subsequent release of interleukin-1α [IL-1α] [45]. Under these circumstances, IL-1α had an anti-apoptotic effect that included activation of IKK ∼18 hr after TNF treatment. Because activated EGF receptors are known to be down-regulated rapidly [58],[59], we hypothesize that in HT-29 cells, EGF inhibits IKK activity following TNF stimulation by inducing EGF receptor down-regulation. This in turn decreases the number of EGF receptors available to transduce autocrine TGFα signaling, a necessity for IKK activation mediated by IL-1α.

From these and similar examples described in Text S1, we conclude that testable biological predictions can be drawn from the logic and connectivity of FL gates including insights that were not apparent from simple inspection of the data.

## Discussion

In this paper we describe the assembly and evaluation of a fuzzy logic model of mammalian signaling networks induced by TNF, EGF, and insulin. The logic gates and their associated membership functions, which encode input-output relationships for interactions among various species in the model, were generated based on study of cellular responses to different cytokine treatments. The gates were then linked together based on prior knowledge of network topology and parameterized using induction or an automatic fitting process that minimized the difference between simulated and experimental trajectories. The resulting models were interpretable with respect to known interactions from the literature, and they generated dynamic trajectories for various signals that were similar to experimental data. We can therefore conclude that efficient assembly of a FL network able to encode complex experimental data is possible.

By building different versions of a FL gate, we were able to intuit potential biological interactions that had gone unnoticed during data mining with other analytic tools. For example, the FL model suggested that MK2 and MEK are co-regulators of ERK. This offers a new explanation for the previously published observation that MK2 has pro-survival effects [21]. Similarly, a link between EGF treatment and IKK inhibition suggests that EGF-induced downregulation of the EGF receptor might interfere with IKK activation by inhibiting TGF-α-induced IL-1α autocrine signaling, which is dependent on EGF receptor activity. Thus, FL modeling yields predictions about the strength and logic of direct and autocrine-indirect processes. In the future, the process of choosing the best FL model can be made more rigorous than what we have undertaken here by automating the fit of rules and membership function to data; this would obviously make the process of extracting hypotheses from models more rigorous.

As a starting point for optimizing FL models, we show that it is possible to fit the rules for individual gates to experimental data. This raises the general possibility that logic-based models can be improved by global fitting procedures [60],[61]. Optimization algorithms such as genetic algorithms and Monte Carlo simulations can be used to fit membership functions and rule weights simultaneously (Figure S2). However, a critical step in optimization of FL models will be the development of objective functions that balance complexity and goodness of fit to data. Because different parameter types encode diverse degrees of freedom, designing a balanced metric will be challenging. Should a model be penalized equally for binary and continuous parameters, or for additional rule weights versus another membership function? Answering these questions will likely require application of theories such as Minimum Description Length and Vapnik-Chervonenkis Theory [62]. These methods employ statistical learning methods (Vapnik-Chervonenkis Theory) or data compression through Turing-style languages (Minimum Description Length) to quantify model complexity. We have already observed that the capacity of multi-state discrete logic gates to effectively capture quantitative data features can be increased by including a greater number of memberships (states) (see Figure S3). Therefore, either fuzzification or inclusion of additional states can strengthen a DL model. A solid metric of model quality would make it possible to compare FL and BL models rigorously as well as evaluate models of the same processes that differ in topology or MFs.

The fuzzy logic framework supports several mechanisms for flexibility including the slope and shape of the membership functions, rule weights, fuzzification and defuzzification procedures, and rule structure. Here, we limited our fuzzification of logic models to a subset of possible FL functions. We used only one degree of membership and one membership shape for entire models and chose the simplest fuzzification algorithms and rule structures. Most of the flexibility in our FL models, as compared to BL models, arose from fuzzy memberships and continuous rule weights that enabled multiple rules to fire simultaneously. By optimizing four variants of the 2-state model (discrete or fuzzy memberships and continuous or binary rule weights, Figure S2), we were able to demonstrate that much of the ability of the FL models to fit the CSR data arose by allowing rule weights to be continuous and not binary. Thus, DL models may be a useful alternative to BL models. If DL models use quantized rather than continuous rule weights, they are likely to achieve a similar flexibility of fuzzified logic models while offering the benefit of faster optimization and easier interpretability with fewer degrees of freedom.

We built models by both manually and automatically fitting model parameters. Though the latter achieved better fits to data, it came at the expense of a loss of model interpretability. Model building methods that balance rigor of automatic optimization with the intuition gained with hand-curated models will be a key step forward. This might be achieved by optimizing quantized rule weights instead of continuous values, or by penalizing models for intermediate weights. Use of a processing algorithm that simplifies sets of optimized rules by excluding those with low weights or merging similar rules would ease the interpretability gap between manually and automatically assembled models. Specialized software that offers a more limited subset of FL capabilities would also streamline model development and improve the computational time required for parameter optimization.

In conclusion, the current FL model of TNF/EGF/insulin-induced signaling in HT-29 cells begins to explore the potential of FL methods to model cell signaling networks. the future, the improvement of automated model fitting, a graphical-user interface tailored to biological applications, and better means to mine and incorporate literature data should facilitate the application of FL modeling methods. Moreover, FL models can be merged with differential equation models to form hybrid models with particular utility in cases in which some processes are well described, receptor-ligand binding and immediate early signaling for example, but the biochemical details of downstream processes such as induced gene transcription are less well specified. One approach to such model fusion would be to reverse engineer part of a differential-equation model to generate the look-up tables necessary for construction of various logic gates. We are currently exploring these and other approaches to expanding the areas of application of FL from industrial control to interpretation of complex biological data.

## Materials and Methods

### Computational programming

Models were written and run using Matlab R2007a. Individual FL gates were constructed and tested using the Matlab Fuzzy Logic Toolbox (Figure S4). Defuzzification was implemented using the Sugeno inference method (“sugeno” in the Fuzzy Logic Toolbox) where for N rules (r) with firing strength s and output level z, the defuzzification is calculated as follows:
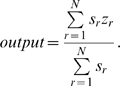



To parameterize the “gauss2mf” membership function shape, a Python script was used to coordinate the MF slope (.250 for FL and .0001 for BL models) with intersections at a 0.5 DOM. Input and output values ranged from 0 to 1 for simplicity and were empirically normalized. Cytokine inputs were scaled non-linearly (see Figure 4) and signals were scaled linearly. Each of the twelve time-steps in the data compendium were equally spaced as inputs to the FL gates even though they were not evenly spaced in real time. Membership functions and input/output ranges could be extended and made nonlinear to reflect absolute time and concentration. We used a default of two states (membership functions) for each variable and the number was increased as needed (heuristically). We decreased the number of free parameters by imposing a single degree of fuzziness on the model and constants for output memberships. The global model was built and run in Simulink, using its standard libraries for the “max” function, time, and time delays. The network is simulated on a synchronous clock (corresponding with the time variable, with a sufficiently small time step) with initial values in downstream gates as 0. Dataset S1 contains the Matlab, Simulink, Fuzzy Logic Toolbox, and Python code used.

### Model fitness

Model fitness was calculated by dividing the sum of the squared difference (RSS) between a model and the data by the degrees of freedom (number of data points-number of parameters for the multi-state models and the number of data points for cross-validation of the 2-state models). For the whole set of simulations, there were 1430 data points. The parameters were counted as fellows: degree of fuzziness (1), MF thresholds (40), and number of unique antecedents (120). The methodology for fitness of the 2-state models is described in Text S1 and Figure S2.

### Global logic gate regression

Rule weight optimization was achieved by using non-linear least squares regression between the model and the dynamic data under ten treatment conditions. Because a gate's output is defuzzified by using a weighted average of the rules fired, sets of firing rules can all have low weights without altering the final output. To highlight firing rules in any circumstance, rule weights were normalized at each iteration of optimization so that the weights of rules with the same antecedents sum to 1. Our manually assembled gates were similar to the fitted gates, but frequently contain condensed and simpler rules sets. For example, we would write the rules “If TNF is L and time is H then MK2 is L” and “If TNF is H and time is H then MK2 is L” in a condensed form: “If time is H then MK2 is L”. Significantly, the condensed form is weighted less heavily in the defuzzification than the explicit form and therefore a balance must be struck between interpretability (for condensed rules) and accuracy (for explicit rules), though we have not encountered misbehavior of logic gates due to condensed rules. For rule fitting, we started by generating full description versions of each possible rule. The optimization procedure was scripted in Matlab R2007a and used the Matlab Optimization toolbox (lsqcurvefit). The Matlab files can be found in Dataset S1. The methodology for global optimization of the 2-state models is described in Text S1 and Figure S2.

## Supporting Information

Text S1 This file contains the text for the Supplementary Materials section.
(0.03 MB DOC)

Figure S1 Fitting MK2 rule weights. (A) A heatmap depicts the data, trained and untrained model with 2 MFs (as described in the main text and Figure 6), and trained and untrained model with 3 MFs for MK2. (B) The fitted rule weights are plotted for the model with 2 MFs (see Figure 6). (C) The regressed rule weights are plotted for the 36 candidate rules. The rules are indicated in tabular format; the first two rows describe the state of the inputs, TNF and time, and the last row is the output MK2 state. L, M, and H represent low, medium, and high states, and E is the state describing the early response lag. Symbols above the plot show whether the rules were present (check), absent (slashed circle), or not applicable (boxed x) in the untrained model. Rules that are different in the trained and the untrained model have red symbols. To compare the optimized rule set with our empirically determined set, the bar graph of rule weights was annotated to indicate discrepancies (red symbols). Seven rules were found to be different, though the differences are easiest understood when grouped into three sets. The first set of rules (“a”) involve the antecedent case “If TNF is M and time is L”. In the untrained model, the output was M while in the trained model, the output was L and H (partial). Therefore, the logic for the trained and untrained model was essentially the same and yielded relatively similar results. In the second set of rules (“b”) for the case “If TNF is H and time if L”, the trained model includes additional outputs of M and L (partial) in addition to H, which is the only rule of the set in the untrained model. The third set of rules (“c”), “If TNF is H or M (partial) and time is M, then MK2 is M”, was missed when the untrained model was built. In comparing the heatmaps of the trained to the untrained model when TNF is H or M and time is M, it is apparent that the untrained model erroneously omitted these rules (A) and the trained model's rules are improvements over the untrained model.
(0.02 MB PDF)

Figure S2 Differences between logic models. (A) A grid differentiates logic models based on differences in uniqueness of rules (whether the rule weights are binary or continuous) and degree of fuzziness in membership functions. Fuzzy logic (FL) models differ from Boolean logic (BL) and discrete multi-state logic (DMSL) models because the membership functions are fuzzy and the rule based need not be unique (e.g. more than one rule can fire for a given input state, even when membership to the input states is discrete). Discrete models (DL) and DMSL models both use discrete membership function but are different in that DL rule bases allow multiple rules to fire (rules are not unique). Roman numerals I–IV map the logic rules to figure (C). The numbers are the averages and standard deviations of the 8-fold cross-validated errors of optimized models of each type. (B) The truth table for the IRS(S) gate described in Figure 2 is expanded to show the case where multiple rules can fire (DL and FL). IRS(S) output values are in bold. One value is gray to reflect its rule weight of 0.25. Where more than one output value is shown, both values result from conflicting firing rules and must be defuzzified. In this case, multiple rule firing results from non-unique rules (overlapping antecedents), not fuzziness in the membership functions. (C) Simulations of the IRS(S) across the spectrum of logic gate-types shown and labeled in (A) are shown with the experimental data (see Figure 5A for cytokine conditions). (D) Non-heatmap representation of globally optimized 2-state FL (IV, blue), DL (II, green), and data (black, shown with the earliest three time points set to their maximum, see above). (E) Non-heatmap representation of globally optimized 2-state model with Fuzzy memberships but binary rule weights (I, blue), BL (III, green), and data (black, shown with the earliest three time points set to their maximum, see above). Because continuous parameters have a higher information capacity than binary parameters, we cannot quantitatively compare BL models with DL or FL models while accounting for the flexibility imparted by their parameters.
(0.05 MB PDF)

Figure S3 Degree of fuzziness in a default 3-state FL model. The FL gates described in the main text were built so that only 2-states (2 MFs) were used when possible. Here, the FL model was built by preferring 3-states per variable. Simulations from 3-state model are plotted (as compared to the data as shown in Figure 5A) for differing degrees of fuzziness [DOFz]. The discrete 3-state model is more able to reproduce the major feature of the data than the DL 2-state model (Figure 5A).
(0.10 MB PDF)

Figure S4 Screen shots illustrating FL gate construction. Screen shots depict the simple graphical user interface used to build the model in the Matlab Fuzzy Logic Toolbox. (A) In the gate set up window, input and output variables are declared. (B) The MFs can be changed graphically by choosing different shapes and altering the MF location and slope. (C–D) The rule editor and viewer is used to write and evaluate rules.
(0.07 MB PDF)

Figure S5 Non-heatmap representation of the data, FL, and DL models. Simulations from the FL model (green) and DL model (blue) are superimposed on the data (black). The layout and conditions are identical to Figure 5A.
(0.02 MB PDF)

Dataset S1 Matlab and python scripts for model simulation and analysis.
(0.08 MB ZIP)
