# Supplementary material for: Fuzzy Logic Analysis of Kinase Pathway Crosstalk in TNF/EGF/Insulin-Induced Signaling
Source: PLoS Comput Biol. 2009 Apr 3;5(4):e1000340. doi: 10.1371/journal.pcbi.1000340 (PMC2663056; doi:10.1371/journal.pcbi.1000340)
Supplement: Text S1 — This file contains the text for the Supplementary Materials section. (0.03 MB DOC) [file pcbi.1000340.s001.doc]

**SUPPLEMENTARY MATERIAL**

**Regulation of IRS-1**

IRS-1 is the canonical adapter protein downstream of the insulin receptor, though some of its many phosphorylation sites are also substrates of other receptor kinases, including EGFR [1].  In modeling IRS-1 phosphorylation at two sites, tyrosine 896 (IRS(Y)) and serine 636 (IRS(S)), we found that both were regulated by TNF and EGF but not by insulin (Figure 3F and 3J).  While the FL gates for IRS(S) and IRS(Y) had the same input variables, the dissimilarity in logic rules revealed differential regulation of the two phosphorylation sites.  Both TNF and EGF treatment positively affect  S636 phosphorylation while TNF inhibits EGF-induced phosphorylation at Y896.  As outlined previously [2], serine phosphorylation at 636 is understood to be downstream of insulin-Akt-mTOR signaling [3,4].  Surprisingly, our model predicts that IRS(S) is unaffected by insulin treatment in HT-29 cells.  Our prediction supports the hypothesis of Gaudet et al.: TNF and EGF induce serine 636 phosphorylation through mTOR, independent of insulin [2].  Further, Gaudet et al. hypothesize that TNF-mediated IRS phosphorylation at serine 636 inhibits EGF-mediated phosphorylation at tyrosine 896 [2]. In the FL gate for IRS(Y), TNF inhibits EGF-induced IRS(Y) activation, supporting this prediction.

**Crosstalk Between ERK and Akt Pathways**

During the formulation of the Akt gate, we included inhibitory crosstalk from ERK to Akt because it has been observed in several experimental systems [5-7].  The introduction of crosstalk greatly simplified the rule-base of the Akt gate, suggesting that this crosstalk exists in the HT-29 cells (Figure 3I).  The mechanism for this crosstalk is not understood in the literature, and our most effective model includes a short time delay from ERK to the Akt gate input.  Negative crosstalk from the ERK to Akt pathways may be the mechanism by which TNF inhibits Akt phosphorylation upon insulin treatment, as observed by Gaudet et al. [2].

**Global Optimization of 2-state Logic Models**

We converted the FL model into a 2-state model by enforcing 2 MFs for each input and output and dividing the weight of the "lost" rules involving intermediate MFs evenly between the two remaining MFs.  Rules were expanded to the explicit form (see Methods).  Rules weights for identical antecedents were then normalized linearly to sum to 1.  To optimize the model, we used the "ga" genetic algorithm in the Optimization Toolbox with MATLAB and custom software for parallelization.  The fitness metric used in optimization was a normalized sum of squared difference between the simulated data and experimental data where the first three time points in the experimental data were replaced each by the maximum value amongst those first three values.  This alteration to the experimental data standard was necessary because a 2-state model cannot model the activation lag immediately following cytokine stimulation.  Normalization was performed by dividing the fitness by the number data points used to calculate the error.

To ensure that the models are not being over-fit, we performed an 8-fold cross-validation by splitting the data into eight non-overlapping, randomly chosen groups and optimized the model by leaving one group out at a time. The cross-validated fitness is calculated as the mean of normalized fitness of the “test” group that omitted during the optimization.

**SUPPLEMENTAL MATERIAL REFERENCES**

1. Lassarre C, Ricort JM (2003) Growth factor-specific regulation of insulin receptor substrate-1 expression in MCF-7 breast carcinoma cells: effects on the insulin-like growth factor signaling pathway. Endocrinology 144: 4811-4819.

2. Gaudet S, Janes KA, Albeck JG, Pace EA, Lauffenburger DA, et al. (2005) A compendium of signals and responses triggered by prodeath and prosurvival cytokines. Mol Cell Proteomics 4: 1569-1590.

3. Harrington LS, Findlay GM, Lamb RF (2005) Restraining PI3K: mTOR signalling goes back to the membrane. Trends Biochem Sci 30: 35-42.

4. Paz K, Hemi R, LeRoith D, Karasik A, Elhanany E, et al. (1997) A molecular basis for insulin resistance. Elevated serine/threonine phosphorylation of IRS-1 and IRS-2 inhibits their binding to the juxtamembrane region of the insulin receptor and impairs their ability to undergo insulin-induced tyrosine phosphorylation. J Biol Chem 272: 29911-29918.

5. Moelling K, Schad K, Bosse M, Zimmermann S, Schweneker M (2002) Regulation of Raf-Akt Cross-talk. J Biol Chem 277: 31099-31106.

6. Yu CF, Liu ZX, Cantley LG (2002) ERK negatively regulates the epidermal growth factor-mediated interaction of Gab1 and the phosphatidylinositol 3-kinase. J Biol Chem 277: 19382-19388.

7. Kiyatkin A, Aksamitiene E, Markevich NI, Borisov NM, Hoek JB, et al. (2006) Scaffolding protein Grb2-associated binder 1 sustains epidermal growth factor-induced mitogenic and survival signaling by multiple positive feedback loops. J Biol Chem 281: 19925-19938.
