# Supplementary figures and images for: Fuzzy Logic Analysis of Kinase Pathway Crosstalk in TNF/EGF/Insulin-Induced Signaling
Source: PLoS Comput Biol. 2009 Apr 3;5(4):e1000340. doi: 10.1371/journal.pcbi.1000340 (PMC2663056; doi:10.1371/journal.pcbi.1000340)

**A**

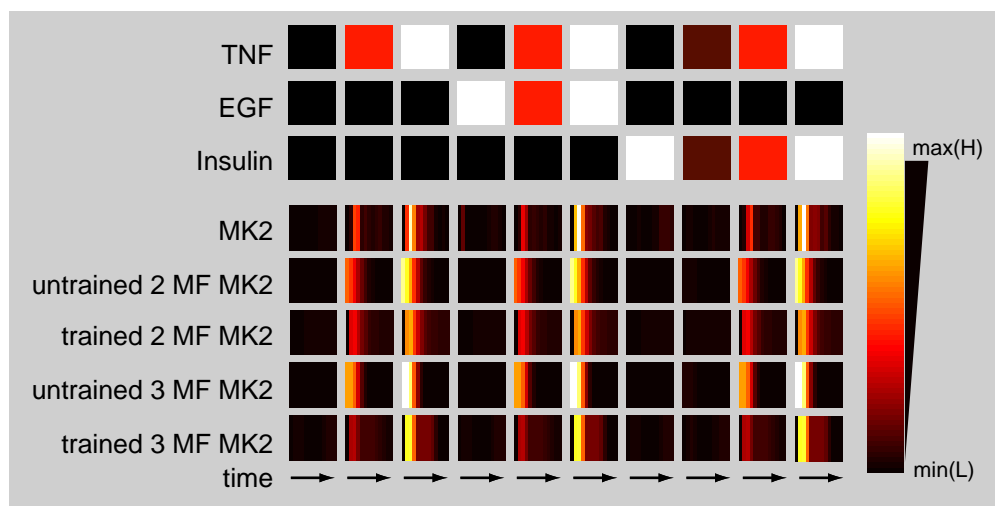

**B**

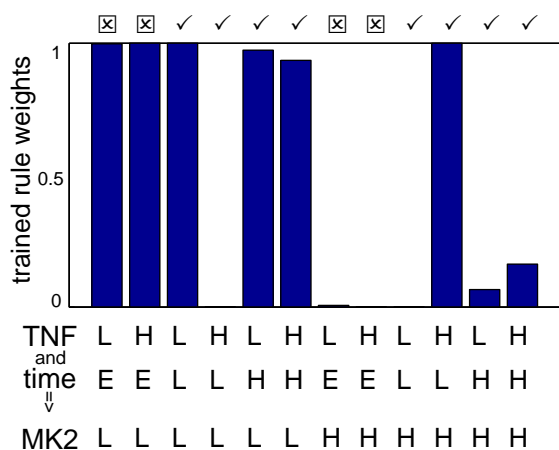

**C**

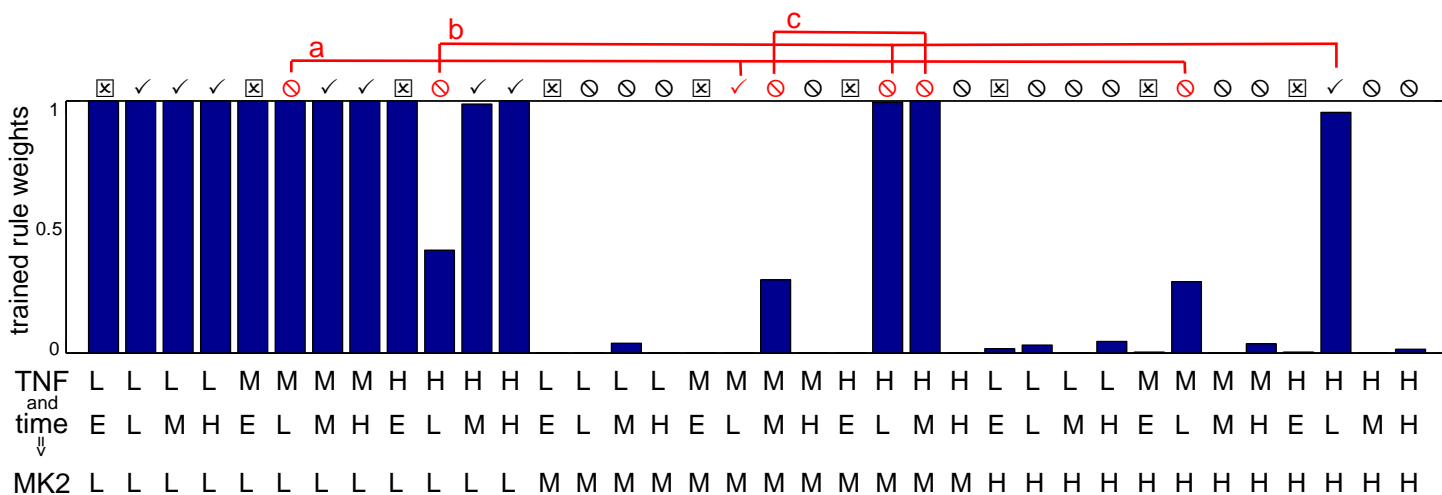

Supplement: Figure S1 — Fitting MK2 rule weights. (A) A heatmap depicts the data, trained and untrained model with 2 MFs (as described in the main text and Figure 6), and trained and untrained model with 3 MFs for MK2. (B) The fitted rule weights are plotted for the model with 2 MFs (see Figure 6). (C) The regressed rule weights are plotted for the 36 candidate rules. The rules are indicated in tabular format; the first two rows describe the state of the inputs, TNF and time, and the last row is the output MK2 state. L, M, and H represent low, medium, and high states, and E is the state describing the early response lag. Symbols above the plot show whether the rules were present (check), absent (slashed circle), or not applicable (boxed x) in the untrained model. Rules that are different in the trained and the untrained model have red symbols. To compare the optimized rule set with our empirically determined set, the bar graph of rule weights was annotated to indicate discrepancies (red symbols). Seven rules were found to be different, though the differences are easiest understood when grouped into three sets. The first set of rules (“a”) involve the antecedent case “If TNF is M and time is L”. In the untrained model, the output was M while in the trained model, the output was L and H (partial). Therefore, the logic for the trained and untrained model was essentially the same and yielded relatively similar results. In the second set of rules (“b”) for the case “If TNF is H and time if L”, the trained model includes additional outputs of M and L (partial) in addition to H, which is the only rule of the set in the untrained model. The third set of rules (“c”), “If TNF is H or M (partial) and time is M, then MK2 is M”, was missed when the untrained model was built. In comparing the heatmaps of the trained to the untrained model when TNF is H or M and time is M, it is apparent that the untrained model erroneously omitted these rules (A) and the trained model's rules are improvements over the u [file pcbi.1000340.s002.pdf]

## Data

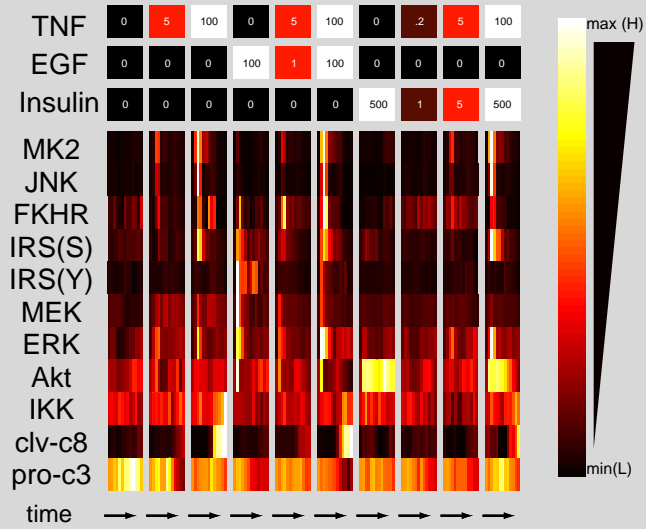

## Model

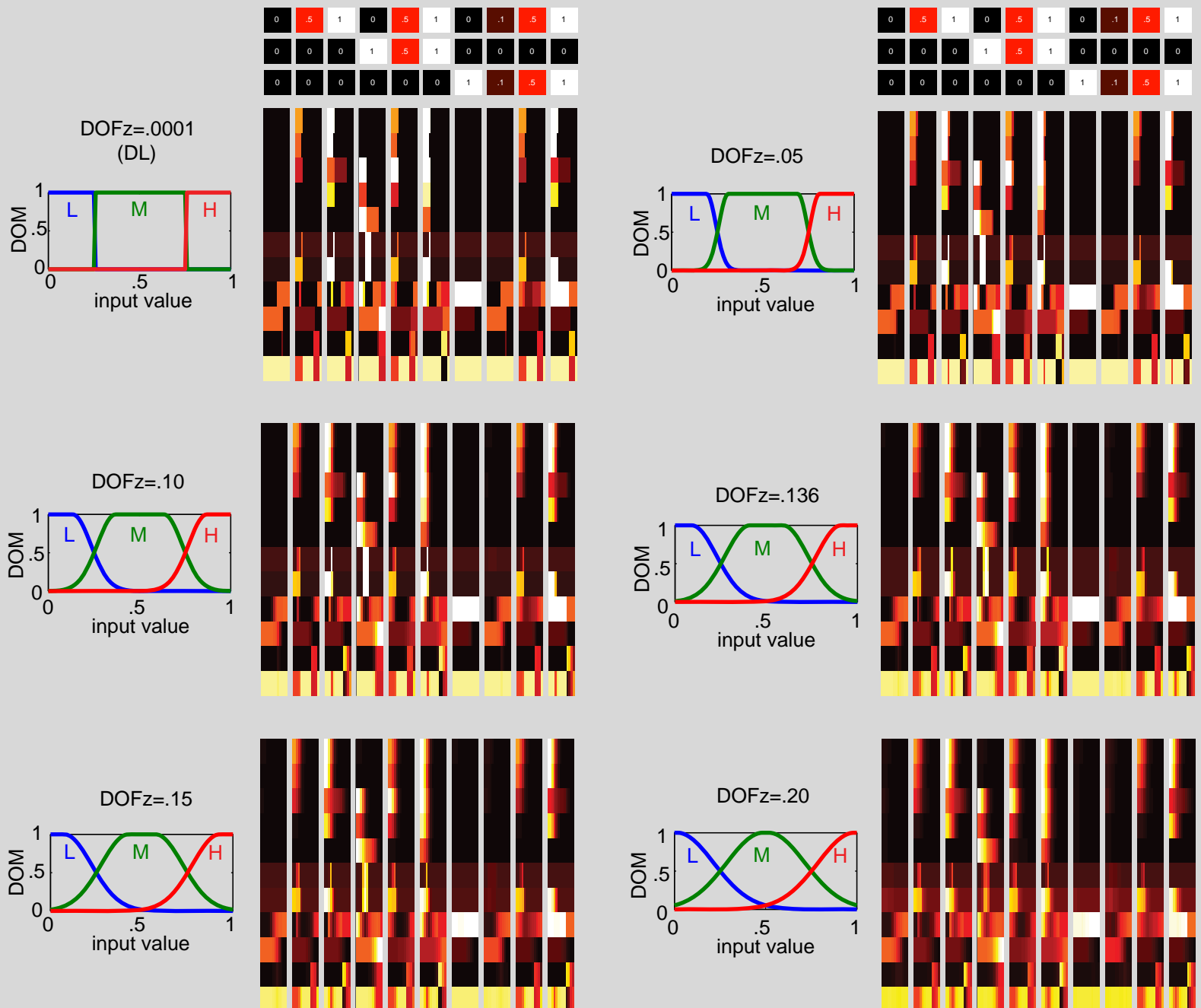

Supplement: Figure S3 — Degree of fuzziness in a default 3-state FL model. The FL gates described in the main text were built so that only 2-states (2 MFs) were used when possible. Here, the FL model was built by preferring 3-states per variable. Simulations from 3-state model are plotted (as compared to the data as shown in Figure 5A) for differing degrees of fuzziness [DOFz]. The discrete 3-state model is more able to reproduce the major feature of the data than the DL 2-state model (Figure 5A). (0.10 MB PDF) [file pcbi.1000340.s004.pdf]

A

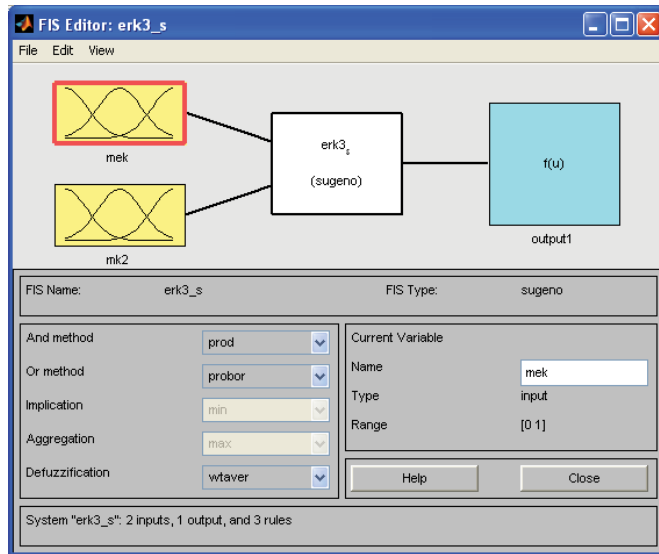

B

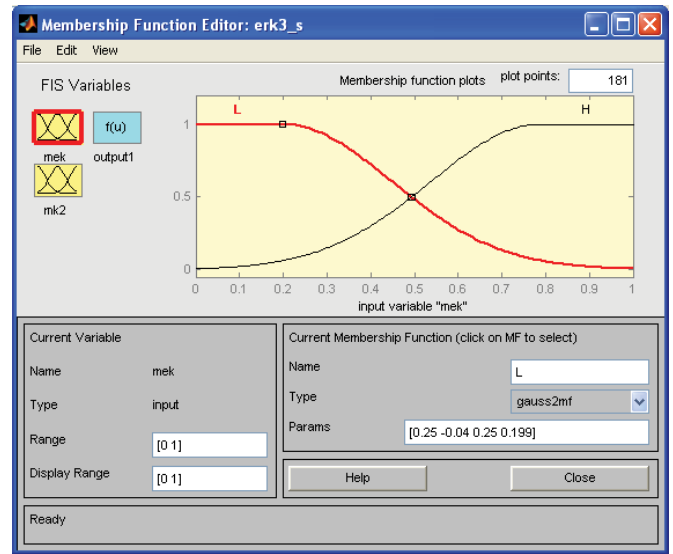

C

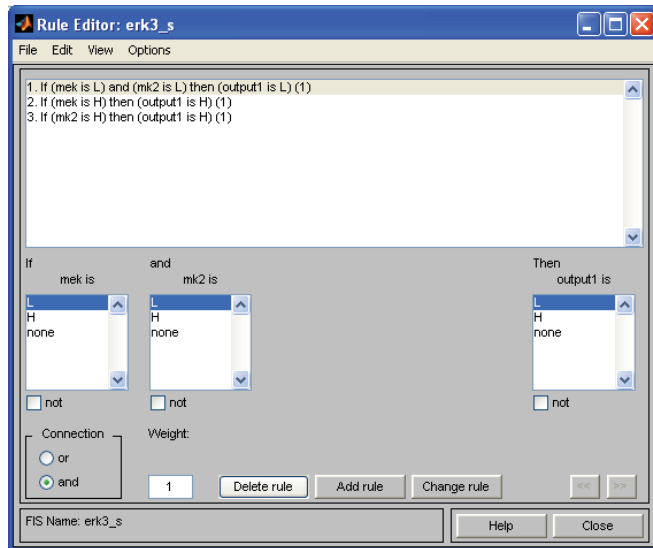

D

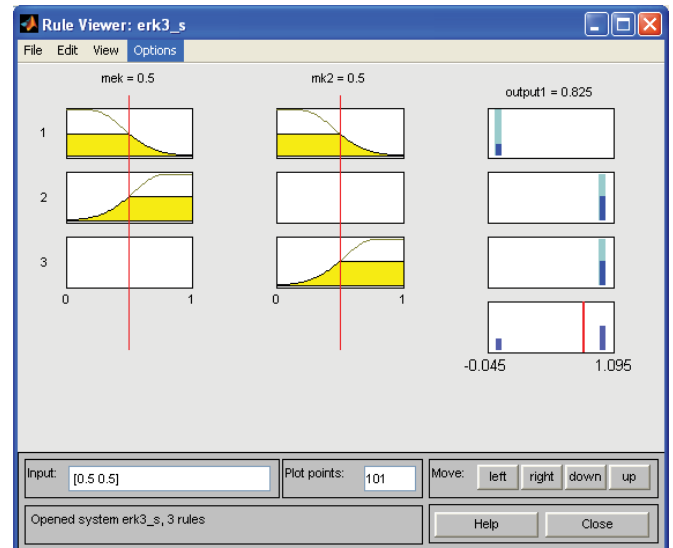

Supplement: Figure S4 — Screen shots illustrating FL gate construction. Screen shots depict the simple graphical user interface used to build the model in the Matlab Fuzzy Logic Toolbox. (A) In the gate set up window, input and output variables are declared. (B) The MFs can be changed graphically by choosing different shapes and altering the MF location and slope. (C–D) The rule editor and viewer is used to write and evaluate rules. (0.07 MB PDF) [file pcbi.1000340.s005.pdf]

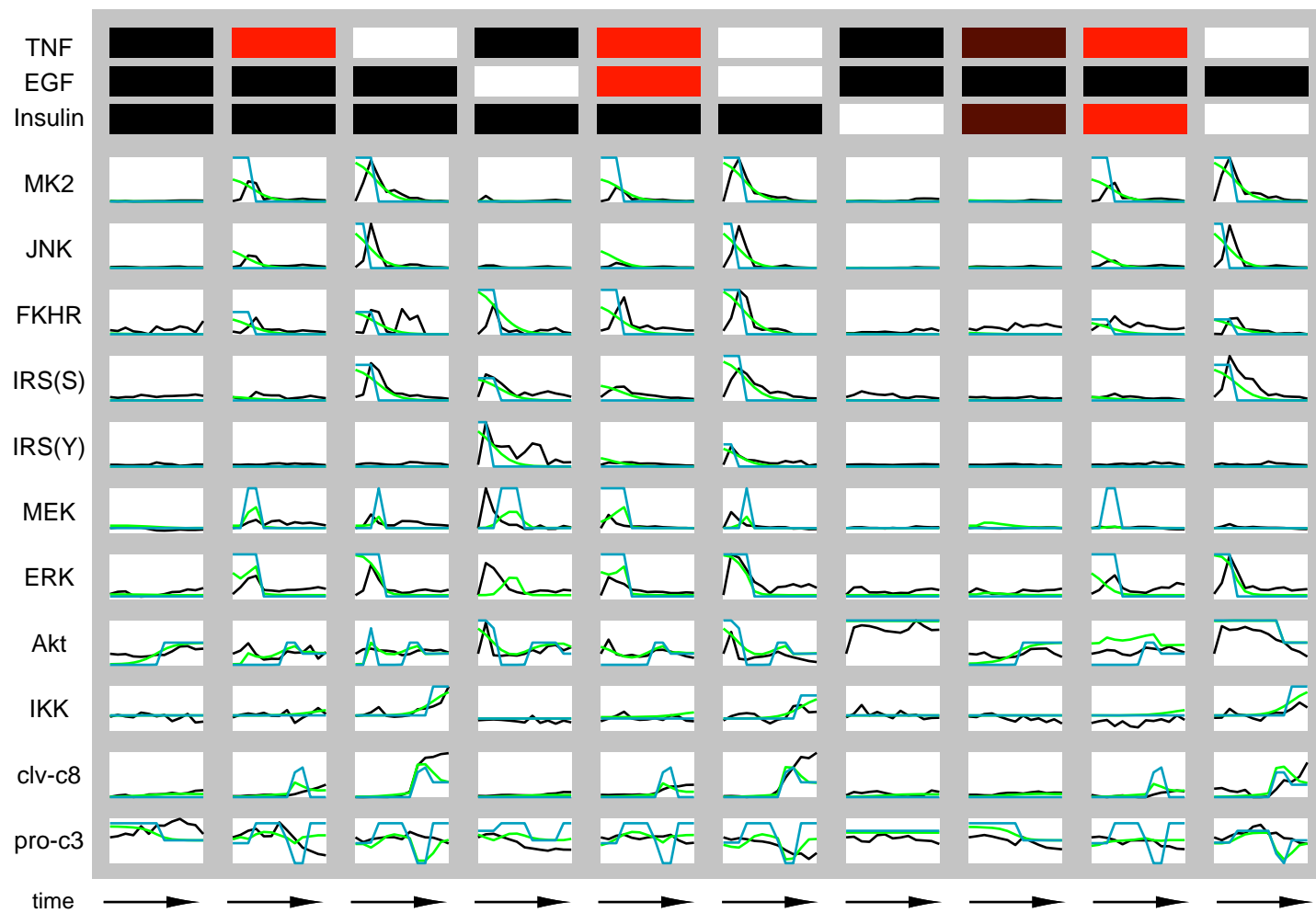

Supplement: Figure S5 — Non-heatmap representation of the data, FL, and DL models. Simulations from the FL model (green) and DL model (blue) are superimposed on the data (black). The layout and conditions are identical to Figure 5A. (0.02 MB PDF) [file pcbi.1000340.s006.pdf]
