# Supplementary material for: Fuzzy Logic Analysis of Kinase Pathway Crosstalk in TNF/EGF/Insulin-Induced Signaling
Source: PLoS Comput Biol. 2009 Apr 3;5(4):e1000340. doi: 10.1371/journal.pcbi.1000340 (PMC2663056; doi:10.1371/journal.pcbi.1000340)
Supplement: Figure S2 — Differences between logic models. (A) A grid differentiates logic models based on differences in uniqueness of rules (whether the rule weights are binary or continuous) and degree of fuzziness in membership functions. Fuzzy logic (FL) models differ from Boolean logic (BL) and discrete multi-state logic (DMSL) models because the membership functions are fuzzy and the rule based need not be unique (e.g. more than one rule can fire for a given input state, even when membership to the input states is discrete). Discrete models (DL) and DMSL models both use discrete membership function but are different in that DL rule bases allow multiple rules to fire (rules are not unique). Roman numerals I–IV map the logic rules to figure (C). The numbers are the averages and standard deviations of the 8-fold cross-validated errors of optimized models of each type. (B) The truth table for the IRS(S) gate described in Figure 2 is expanded to show the case where multiple rules can fire (DL and FL). IRS(S) output values are in bold. One value is gray to reflect its rule weight of 0.25. Where more than one output value is shown, both values result from conflicting firing rules and must be defuzzified. In this case, multiple rule firing results from non-unique rules (overlapping antecedents), not fuzziness in the membership functions. (C) Simulations of the IRS(S) across the spectrum of logic gate-types shown and labeled in (A) are shown with the experimental data (see Figure 5A for cytokine conditions). (D) Non-heatmap representation of globally optimized 2-state FL (IV, blue), DL (II, green), and data (black, shown with the earliest three time points set to their maximum, see above). (E) Non-heatmap representation of globally optimized 2-state model with Fuzzy memberships but binary rule weights (I, blue), BL (III, green), and data (black, shown with the earliest three time points set to their maximum, see above). Because continuous parameters have a higher information capacity than bin [file pcbi.1000340.s003.pdf]

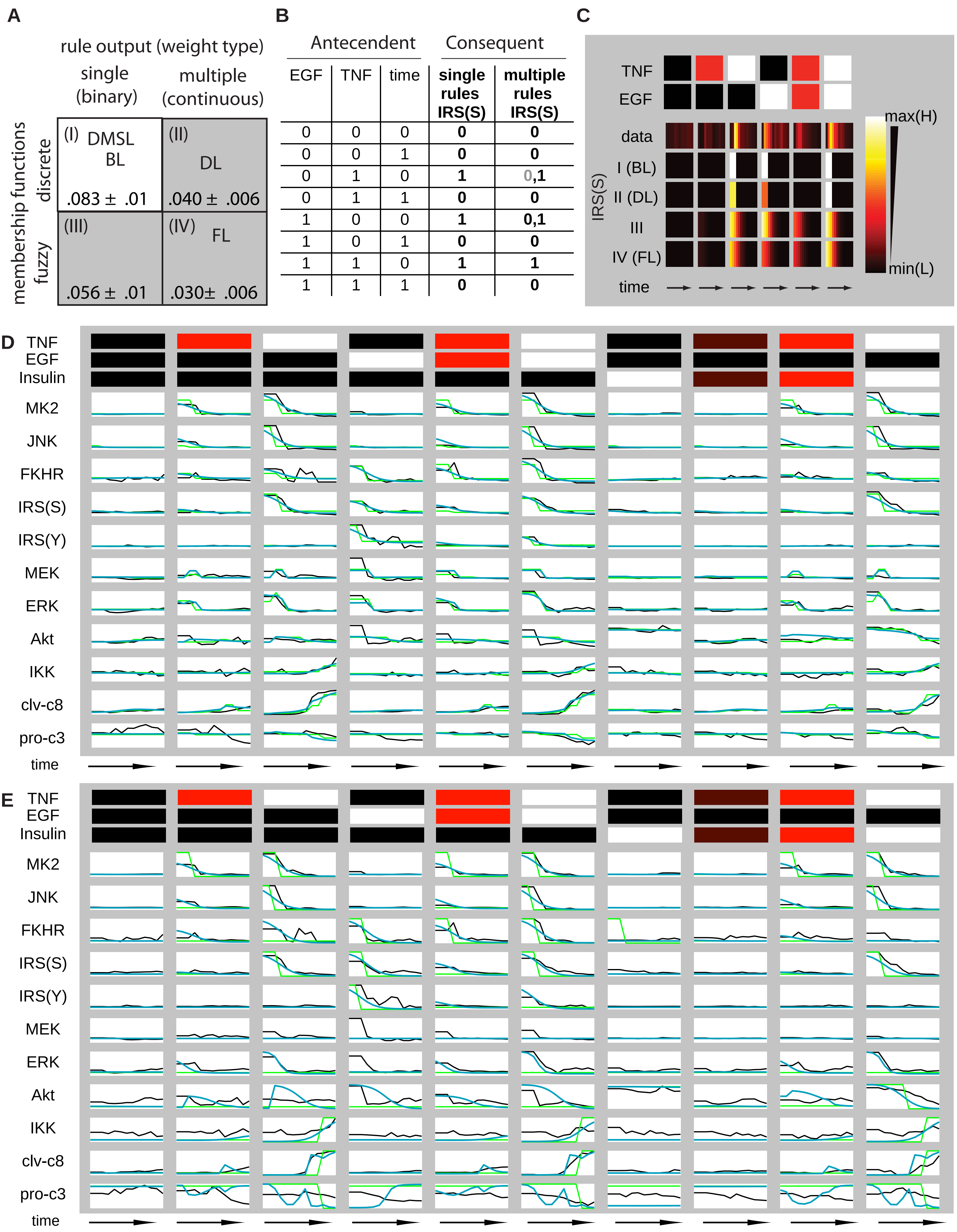
A
rule output (weight type)
single (binary)
multiple (continuous)
membership functions
discrete
fuzzy
(I) DMSL BL .083 ± .01
(II) DL .040 ± .006
(III) .056 ± .01
(IV) FL .030± .006
B
Antecendent
Consequent
EGF
TNF
time
single rules IRS(S)
multiple rules IRS(S)
0 0 0 0 0
0 0 1 0 0
0 1 0 1 0,1
0 1 1 0 0
1 0 0 1 0,1
1 0 1 0 0
1 1 0 1 1
1 1 1 0 0
C
TNF
EGF
data
IRS(S)
I (BL)
II (DL)
III
IV (FL)
time
max(H)
min(L)
D
TNF
EGF
Insulin
MK2
JNK
FKHR
IRS(S)
IRS(Y)
MEK
ERK
Akt
IKK
clv-c8
pro-c3
time
E
TNF
EGF
Insulin
MK2
JNK
FKHR
IRS(S)
IRS(Y)
MEK
ERK
Akt
IKK
clv-c8
pro-c3
time
